# Influence of Phthalates on *in vitro* Innate and Adaptive Immune Responses

**DOI:** 10.1371/journal.pone.0131168

**Published:** 2015-06-25

**Authors:** Juliana Frohnert Hansen, Claus Henrik Nielsen, Marianne Møller Brorson, Hanne Frederiksen, Marie-Louise Hartoft-Nielsen, Åse Krogh Rasmussen, Klaus Bendtzen, Ulla Feldt-Rasmussen

**Affiliations:** 1 Department of Medical Endocrinology, PE 2132, Rigshospitalet, University of Copenhagen, Copenhagen, Denmark; 2 Institute for Inflammation Research, Section 7521, Rigshospitalet, University of Copenhagen, Copenhagen, Denmark; 3 Department of Growth and Reproduction, Rigshospitalet, University of Copenhagen, Copenhagen, Denmark; Harvard Medical School, UNITED STATES

## Abstract

Phthalates are a group of endocrine disrupting chemicals, suspected to influence the immune system. The aim of this study was to investigate the influence of phthalates on cytokine secretion from human peripheral blood mononuclear cells. *Escherichia coli* lipopolysaccharide and phytohemagglutinin-P were used for stimulation of monocytes/macrophages and T cells, respectively. Cells were exposed for 20 to 22 hours to either di-ethyl, di-n-butyl or mono-n-butyl phthalate at two different concentrations. Both diesters were metabolised to their respective monoester and influenced cytokine secretion from both monocytes/macrophages and T cells in a similar pattern: the secretion of interleukin (IL)-6, IL-10 and the chemokine CXCL8 by monocytes/macrophages was enhanced, while tumour necrosis factor (TNF)-α secretion by monocytes/macrophages was impaired, as was the secretion of IL-2 and IL-4, TNF-α and interferon-γ by T cells. The investigated phthalate monoester also influenced cytokine secretion from monocytes/macrophages similar to that of the diesters. In T cells, however, the effect of the monoester was different compared to the diesters. The influence of the phthalates on the cytokine secretion did not seem to be a result of cell death. Thus, results indicate that both human innate and adaptive immunity is influenced *in vitro* by phthalates, and that phthalates therefore may affect cell differentiation and regenerative and inflammatory processes *in vivo*.

## Introduction

Phthalates are a group of endocrine disrupting chemicals (EDCs), with hundreds of million tons produced each year [1]. Phthalates added during the manufacturing process are not chemically bound to the end-products and leach easily into the environment. Exposure to humans and wildlife cannot be avoided due to their ubiquitous presence [2–4]. Phthalates are produced by the industry as lipophilic diesters, which are rapidly metabolised to their respective monoester. Large phthalate molecules are further metabolised to oxidative metabolites [5,6].

Di-n-butyl phthalate (DnBP) and di-ethyl phthalate (DEP) are two common phthalates with measurable metabolites in the urine of both children and adults [7]. DnBP is used mainly as a plasticizer but also as an additive in cosmetics, floor carpets [8,9] and in coating of enteric medication [10]. DEP is commonly used in cosmetic products but also in plastic packaging and cellulose plastic films [11]. DnBP has been shown to have adverse effects on especially the male reproductive system [1], and its use in childrens’ toys is prohibited in the European Union [12,13]. DEP is believed to be less toxic and thus not restricted to the same extent as DnBP is [13,14], though more recent studies have found DEP to influence reproduction endpoints as much as DnBP does [15,16].

Epidemiological studies have suggested a positive association between phthalate exposure and allergy or asthma, [17,18]. Several *in vivo* and *in vitro* studies have investigated the influence of phthalates on immunoglobulin secretion, Th1/Th2 differentiation and cytokine secretion [18], though studies on the influence of cytokine secretion by primary human peripheral blood mononuclear cells (MNC) are very few [19,20].

Cytokine secretion by MNC is influenced by prostaglandin (PG), presumably via the second messenger 3'-5'-cyclic adenosine monophosphate (cAMP) [21]. Phthalate structure resembles that of PGs, and the ability of phthalates to influence PG signalling and synthesis has been studied in diverse cell cultures [22–27]. In mast cells, DnBP inhibited dose-dependently Prostaglandin D2 (PGD2) synthesis [22], and in bone marrow B cells, proliferation and apoptosis were inhibited synergistically by mono-(2-ethylhexyl) phthalate (MEHP) and 15-deoxy-prostaglandin J_2_ [24]. However, the influence of phthalates on PG-secretion and signalling has not yet been studied in human MNC.

As phthalate metabolism in human cell cultures [22,28,29] and effects of these EDCs on selective cellular processes in human MNC are poorly studied [19,20], the aim of the present study was to investigate if DEP, DnBP and its monoester, mono-n-butyl phthalate (MnBP), were able to influence cytokine secretion by human immune cells. To this end, cultures of MNC stimulated with *E*. *coli* lipopolysaccharide (LPS) or phytohemagglutinin-P (PHA-P) were used. The involvement of PG-signalling in this process, as well as MNC ability to metabolise the investigated phthalates was also examined.

## Methods and Materials

### Subjects

Blood samples were drawn from seven healthy female and male staff members at the Department of Medical Endocrinology, PE 2132, Rigshospitalet. Fresh blood was collected in sodium-heparin tubes (Vacutainer, Becton Dickinson, Franklin Lakes, NJ) 30 to 60 minutes prior to isolation of MNC.

### Cell cultures

MNC were isolated by density centrifugation (Ficoll-Hypaque, Almeco, Esbjerg, Denmark) and Lymphoprep (Axis-Shield, Oslo, Norway), washed three times in phosphate-buffered saline without calcium and magnesium (PBS), and re-suspended in Hams F12 growth medium with Glutamax, (both purchased from Gibco, Invitrogen, Thermo Fischer Scientific, Waltham, MA, USA), and supplemented with 5% (v/v) foetal bovine serum (Biological Industries, Beit HaEmek, Israel), non-essential amino acids and penicillin/Streptomycin (Gibco, Invitrogen) to a concentration of 10^6^ cells per ml. The cell suspension was distributed into three 24-well polystyrene plates (NUNC, Roskilde, Denmark) with 10^6^ cells per well. Subsequently, DEP (CAS 84-66-2), DnBP (CAS 84-74-2) or MnBP (CAS 131-70-4) (Sigma-Aldrich, St. Louis, MO, USA) were added to final concentrations of 0.1 and 100 μM. After cultivation for one hour at 37°C and 5% CO_2_, *E*. *coli* LPS or PHA-P (CAS 9008-97-3) (both from Sigma-Aldrich) was added at final concentrations of 100 pg/ml and 5μl/ml, respectively. Unstimulated MNC, i.e. cell cultures exposed to phthalates but without LPS or PHA-P stimulation, were also investigated. MNC cultures were further incubated for 20 to 22 hours at 37°C, after which cell supernatants were harvested after centrifugation at 15000 x G for 5 min and stored at 4°C until analysis. All experiments were performed in duplicates. Since phthalates were dissolved in ethanol prior to further dilution in culture media, two controls were included in all experiments: culture medium-control and culture medium-control with 1 ‰ (v/v) ethanol added. Only the latter was used for statistical analysis.

### Measurement of phthalate metabolites

The primary metabolites of DEP and DnBP (mono-ethyl phthalate (MEP) and mono-n-butyl phthalate (MnBP), respectively), were quantified in cell supernatants by isotope-diluted online-TurboFlow-liquid chromatography-tandem mass spectrometry as previously described [30], but modified to an 11.5 min runtime. Inter- and intra-assay validation of cell media spiked with MEP and MnBP, at four different levels, showed standard deviations from 2.9% to 8.8%. Growth medium and PBS were also examined for presence of above mentioned phthalate metabolites.

### Cytokine assessments

Cytokine levels were measured in cell culture supernatants using cytometric bead array (CBA) kits, HumanTh1/Th2 Cytokine Kit II and Human Inflammation Kit (Becton Dickinson, Franklin Lakes, NJ, USA), according to the manufacture’s protocols with the exception that threefold higher dilutions of all reagents were used. In brief, the Th1/Th2 kit measures interleukin (IL)-2, IL-4, IL-6, IL-10, tumour necrosis factor (TNF)-α and interferon (IFN)-γ, whereas the inflammation kit measures IL-1β, IL-6, CXCL8, IL-10, IL-12 and TNF-α. The samples were analysed in a FACSCalibur flow cytometer (Becton Dickinson). As stated by the manufacturer, the respective intra- and inter-assay variations (% CV) were 2–5% and 3–11% for the Th1/Th2 kit, and 2–10% and 4–15% for the inflammation kit. The standard range in both assays was 20 to 5000 pg/ml.

### cAMP assessment

The contents of cAMP in supernatants were tested by adding 3-Isobutyl-1-methylxanthine (IBMX) concurrently with the phthalates. IBMX was diluted in ethanol (final ethanol concentration 1%), thus the controls in these experiments contained 1.1% ethanol. Cells were harvested as described above, and the cAMP concentration was assessed by a competitive protein binding method [31]. The intra-assay variation of 0.4 and 1.4 μM was 4.7 and 7.2%, respectively (n = eight duplicates for each control level) and the inter-assay variation was 11.6% (n = five low and five high cAMP-concentration-samples in duplicates, range 0.29–0.45 μM and 1.10–1.71 μM, respectively). The standard range was 0.05 to 2.0 μM.

### Toxicity assessment

Phthalate-induced cytotoxicity was analysed by testing the content of lactate dehydrogenase (LDH) in cell culture supernatants. A homogenous membrane integrity assay (CytoTox-ONE, Promega, Fitchburg, WI, USA) was used according to the manufacture’s protocol with the modification that the LDH content was assessed in harvested supernatants, instead of directly in cell cultures. Briefly, a pilot study was performed using freshly harvested MNC supernatants from one of the experiments which included positive controls exposed to lysis solution (9% Triton X-100, included in CytoTox-ONE assay). Final concentration of Triton X-100 in positive controls was 0.02, 0.2 or 1.9 mg/l. Hereafter, analysis was performed in supernatants from cells stored at -20°C without positive controls. Frozen cell supernatants were thawed, and transferred to a black 96-well half area micro plate (Th.Geyer, Renningen, Gerrmany) followed by addition of the same volume CytoTox-One reagent to all wells. The micro plate was stirred in a shaker and incubated at room temperature for 10 to 15 minutes. Hereafter, stop solution was added, and the plate was again shaked before results were read on a fluorometer (Victor2, PerkinElmer, Waltham, MA, USA). The LDH contents/amount of lysed cells was proportional to the fluorescence produced and given in relative fluorescence units (RFUs), as a measure of toxicity of the phthalates.

### Endotoxin test

Prior to use in the experiments, all reagents were tested for endotoxin content using the Limulus Amebocyte Lysate (LAL) QCL-1000 assay (Lonza, Basel, Switzerland). If reagents tested positive, endotoxin was removed by Triton X-114, as described earlier [32].

### Ethics

The study was approved by The Danish committees on Health Research Ethics, Capital region (Protocol number: H-1-2012-110), which in Denmark/Copenhagen also functions as the institutional review board. According to the committee law by the Danish Committees on Health Research Ethics, neither written nor oral informed consent is needed in studies on anonymous human blood samples. Blood samples of this study were drawn from anonymous healthy human volunteers, whose identity was unknown to the investigators, and thus no informed consent was obtained.

### Statistical analysis

Cytokine secretion (mean of duplicates) was analysed by two way ANOVA followed by Tukey’s post hoc analysis. Cytokine secretion in the two controls were analysed by paired t-test. P-values <0.05 were considered statistically significant.

## Results

### Endotoxin contents in reagents

PHA-P contained high concentrations of endotoxins, which were successfully removed, and results from the SDS-PAGE suggested that PHA-P was unchanged by this endotoxin removal. The endotoxin contents in all other reagents used in this study were below the detection limit of the LAL assay.

### Metabolism of phthalates in MNC

Both DEP and DnBP were metabolised in MNC to their respective monoesters (MEP and MnBP), and all of the added MnBP was recovered as the monoester (n = one cell culture, unstimulated, in single determination, data not shown), demonstrating no further metabolism.

The amount of diester metabolised by the cells varied for DEP and DnBP. Hundred μM of DEP was metabolised to 120 μM of MEP (n = one well in single determination), but 100 μM of DnBP was metabolised to 61 μM MnBP (n = one well in single determination). When added at lower concentration (0.1 μM), all DEP and DnBP seemed to be metabolised to their respective monoester, with measured concentrations 0.5 and 0.1 μM, respectively (data not shown).

Notably, both MEP and MnBP were detected in wells adjacent to those to which the respective diester had been added. Thus, MEP was detected at a concentration of 2.8 μM in the closest adjacent well to the experiment well, where 100 μM DEP had been added. MnBP at a concentration of 0.4 μM was detected in the closest adjacent well to the experiment well, where 100 μM DnBP had been added. The respective MEP and MnBP content in the culture media was 0.0 and 0.5 nM. No detectable levels were found in PBS (data not shown).

### Cytokine secretion from unstimulated cells

Supernatant levels of cytokines (IL-1β, IL-2, IL-4, IL-6, CXCL8, IL-10, TNF-α, and IFN-γ) from unstimulated MNC were in most wells below the lowest standard (20 pg/ml), and all were far below supernatant levels of stimulated cells, except for CXCL8. Median CXCL8 level and range in culture medium- and ethanol-control were 126 (48–513) pg/ml and 133 (59–3135) pg/ml, respectively (n = seven cultures in duplicates). Compared to other cytokines, the measured CXCL8 concentrations from unstimulated cells had high variance, and were thus interpreted as unspecific activation of MNC.

### Cytokine secretion from LPS-stimulated MNC

To examine the influence of phthalates on cells of the innate immune system, MNC were stimulated with *E*. *coli* LPS. LPS activates human monocytes/macrophages through Toll-like receptor 4. Human B cells present in MNC may contribute to cytokine production, but this is considered secondary to LPS-activation of monocytes/macrophages [33]. Nonetheless, LPS activates B cells in an antigen-nonspecific manner, and a possible cytokine response from B cells in MNC is therefore due to innate functions of B cells.

LPS stimulation induced secretion of IL-1β, IL-6, CXCL8, IL-10 and TNF-α from MNC (Table 1), but not IL-12 (data not shown). Cytokine secretion differed between the negative controls in that it was higher in the ethanol- than in the medium-control (IL-10: p = 0.008; IL-1β: p = 0.04), except for IL-6 (p = 0.26), CXCL8 (p = 0.08) and TNF-α (p = 0.25); n = 7 cultures in duplicates (data not shown).

**Table 1:** Median and range of ethanol controls from LPS- and PHA-P-stimulated MNC (n = 7 and 6 cultures in duplicates, respectively).

Stimulation	Cytokine	Median (pg/ml)	Range (pg/ml)
**LPS**	IL-1β	653	63–1110
	IL-6	6083	4670–9905
	CXCL8	57133	27325–87093
	IL-10	84	35–156
	TNF-α	564	92–1757
**PHA-P**	IL-2	1169	459–3333
	IL-4	72	26–201
	IL-6	5226	3340–14133
	IL-10	209	65–358
	TNF-α	1894	791–4588
	IFN-γ	1897	844–5685

LPS: lipopolysaccharide. PHA-P: phytohemagglutinin-P.

**Fig 1:**
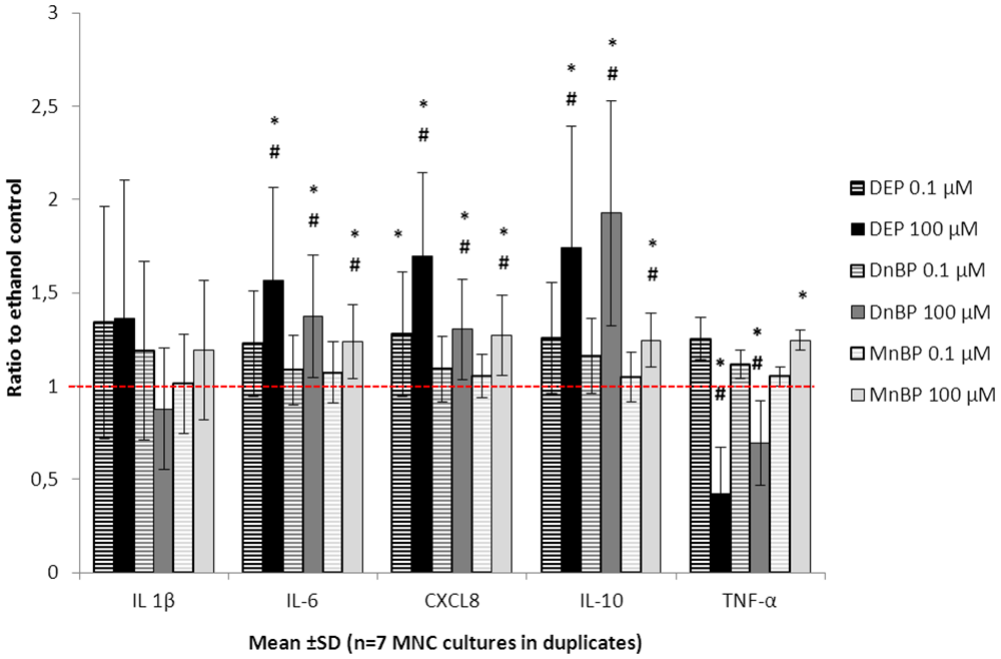
Influence of phthalates on the cytokine response of innate immune cells. MNC cultures were stimulated with 100 pg/ml E. coli LPS and exposed to di-ethyl phthalate (DEP), di-n-butyl phthalate (DnBP), or mono-n-butyl phthalate (MnBP), at two different concentrations, for 20–22 hours. The resulting production of IL-1β, IL-6, CXCL8, IL-10 and TNF-α are shown as ratio to the respective ethanol control. The red dashed line indicates the level of the ethanol controls (ratio = 1). * = p<0.05 compared to ethanol control, # = p<0.05 compared to low phthalate exposure (0.1 μM).

None of the investigated diesters nor the monoester were found to have any influence on IL-1β secretion by MNC. On the other hand, secretion of IL-6, CXCL8, IL-10 and TNF-α was significantly influenced by both diesters and by the monoester (Fig 1 and S1 Table). DEP and DnBP enhanced secretion of IL-6, CXCL8 and IL-10, but inhibited TNF-α secretion. The monoester, MnBP, enhanced the secretion of all four cytokines.

A statistically significant dose-response relationship was found only for DEP on the CXCL8 secretion (estimated differences and 95% confidence intervals in S1 Table).

### Cytokine secretion from PHA-P-stimulated MNC

**Fig 2:**
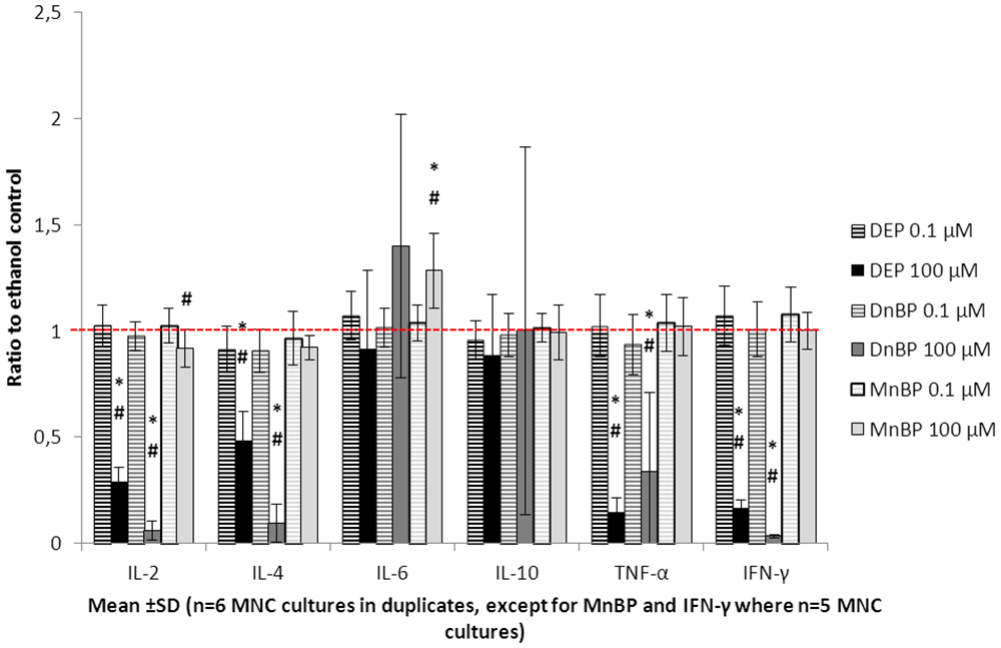
Influence of phthalates on T-cell responses. MNC cultures were stimulated with phytohemagglutinin-P and exposed to di-ethyl phthalate (DEP), di-n-butyl phthalate (DnBP) or mono-n-butyl phthalate (MnBP), at two different concentrations, for 20–22 hours. The resulting production of IL-2, IL-4, IL-6, IL-10, TNF-α and IFN-γ are shown as ratios to the respective ethanol control. The red dashed line indicates the level of the ethanol controls (ratio = 1). * = p<0.05 compared to ethanol control, # = p<0.05 compared to low phthalate exposure (0.1 μM).

To examine the influence of phthalates on T cells of the adaptive immune system, MNC were stimulated with PHA-P. This increased the production of IL-6, IL-10 and TNF-α, as well as of the more T cell specific cytokines IL-2, IL-4 and IFN-γ (Fig 2). There was no difference in cytokine secretion between the ethanol and culture medium-negative controls (IL-2: p = 0.99, IL-4: p = 0.89, IL-6: p = 0.96, IL-10: p = 0.71, TNF-α: p = 0.26, IFN-γ: p = 0.75) (n = 6 cultures in duplicates, data not shown).

PHA-P-induced secretion of IL-2, IL-4, TNF-α and IFN-γ was significantly impaired by both DEP and DnBP (Fig 2 and S2 Table). However, IL-6 and IL-10 secretion was unaffected by the two diesters. MnBP enhanced IL-6 secretion and tended to inhibit IL-2 secretion from PHA-P-stimulated MNC, but had no influence on the other measured cytokines.

There were no significant dose response relationships for any of the tested cytokines. Only the high phthalate concentration (100 μM) had a significant influence on cytokine secretion by PHA-P-stimulated MNC (estimated differences and 95% confidence intervals in S2 Table).

### cAMP contents in supernatants of phthalate-exposed cells

Due to the structural resemblance between phthalates and PGs [25], we investigated if phthalates mediated their influence on cytokine secretion through the second messenger of PGs, cAMP, the content of which was assessed in supernatants from an LPS-stimulated MNC culture. The cultures were exposed to DEP, DnBP and MnBP, all at concentrations of 0.1 and 100 μM. No cAMP was detected in controls, and neither DEP, DnBP nor MnBP were able to induce a measurable cAMP secretion (n = one MNC culture in duplicates, data not shown).

### Cytotoxicity of phthalates

**Fig 3:**
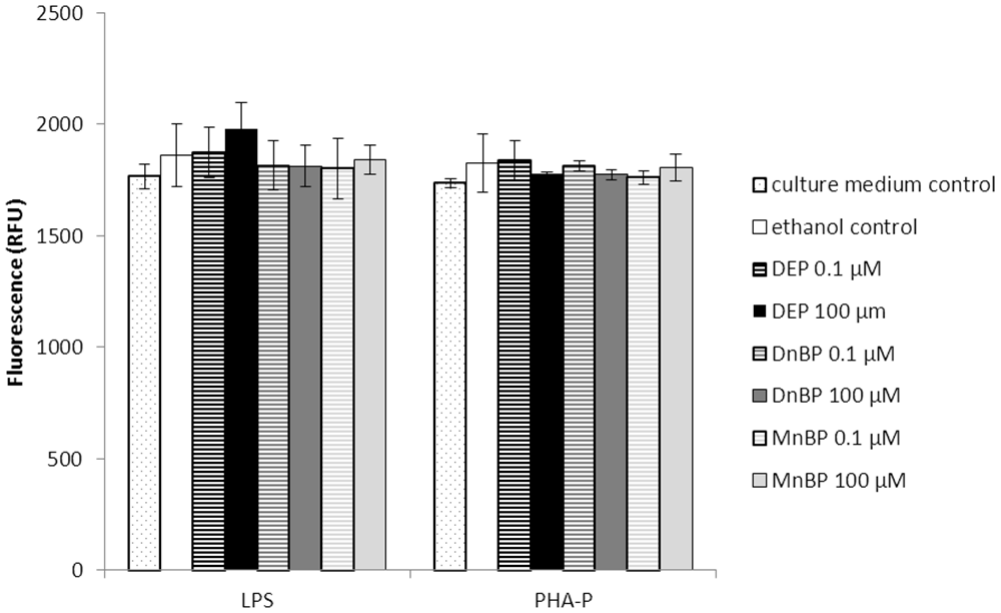
LDH contents (y-axis) in supernatants from LPS- and PHA-P-stimulated MNC exposed to phthalates. The LDH content was proportional to the produced fluorescence (given in relative fluorescence units (RFUs)).

To evaluate if changes in the MNC supernatant cytokine content were caused by leakage of cytokines from dying cells, the LDH concentration, as a measure of leaked proteins, was assessed in culture supernatants. Lysis of MNC, serving as a positive control, showed a concentration-dependent increase in LDH levels at three concentrations of Triton X-100 (0.02, 0.2 or 1.9 mg/l), which were respectively 19, 20 and 38% higher than the medium-control from the same MNC (n = one unstimulated culture in triplicates, data not shown). LDH levels in the ethanol control from both LPS- and PHA-P-stimulated MNC were slightly higher than the respective culture medium control (Fig 3). Only LPS-stimulated MNC exposed to DEP at 100 μM had increased LDH levels compared to the ethanol control. This was, however, not observed in the PHA-P-stimulated MNC, where LDH levels in the supernatants from phthalate-exposed cells were similar to those from the ethanol control (Fig 3).

To determine cell viability in a classical manner, all cell cultures were inspected by light microscopy just before the supernatants were harvested. MNC exposed to Triton X-100 at 0.2 or 1.9 mg/l showed a marked concentration-dependent increase in visible dead cells, while control and phthalate-exposed MNC remained unchanged (data not shown). In PHA-P-stimulated MNC, cells tended to cluster together in both phthalate-exposed and control cells, an observation not made in unstimulated or LPS-stimulated MNC.

## Discussion

The two phthalate concentrations used in this experiment were chosen to simulate exposure doses in humans. The high concentration used in this study (100 μM) is a possible exposure for the general population but especially for hospitalised or other exposed individuals. Urinary concentrations of MEP up to 13 mg/l (67μM) were found in a population of Danish healthy adolescents [7]. Preterm infants in Finland had measurable urinary DEHP metabolites up to 46 mg/l and MnBP concentrations up to 6.5 mg/l (29 μM) [34]. Patients taking medication that are contain DnBP in the coating, have even higher urinary MnBP concentrations (42 mg/l i.e. 189 μM of MnBP) [35]. The low concentration, 0.1 μM (or 22.2 μg/l for MnBP) was about the same, or even lower than the median monoester concentration excreted in urine [7,12]. An influence by phthalates in this study was almost exclusively observed at 100 μM and more concentrations in between 0.1 and 100 μM would be preferable in future studies to confirm that the influence is indeed a dose-response effect.

MNC were found to metabolise both DEP and DnBP to their respective monoester. However, when added at the high concentration (100 μM), not all of the DnBP was metabolised to its monoester, unlike what was observed for DnBP added at a lower concentration (0.1 μM), and for DEP at both concentrations (100 and 0.1 μM). This indicates that uptake and metabolism of phthalates by the cells is a saturable process, at least for some phthalates. DnBP has a higher molecular weight with longer alkyl side chains than DEP (278 versus 222 g/mol), and might be taken up less easily than DEP. Another possibility is that DnBP was not available to cells to the same extent as was DEP. DnBP has a lower solubility in water than DEP, 10 mg/l [9] versus 1080 mg/l [11], and both diesters were dissolved in ethanol prior to further dilution in culture media. DnBP, however, was close to the water solubility limit (27.8 mg/l) when added at 100 μM to the cell cultures, and might not have been fully dissolved.

Detection of measurable monoesters in wells adjacent to those supplied with the respective diesters could be a consequence of using phthalate-contaminated laboratory equipment, though this was sought avoided in our laboratory. Another possibility is phthalate migration on culture plates, a pitfall previously observed in cultures of primary human thyroid cells (Hansen et al., unpublished). Regardless of the cause, unintended contamination represents a potential bias, since cells may not be exposed to single phthalates but rather to a mixture of phthalates. In recent years, investigators have become aware of the synergistic influence of low dose EDCs acting together, known as the “cocktail effect” [36]. It is impossible to say whether or not cytokine secretion might have been affected by phthalate mixtures in our experiments.

DEP added at 0.1 μM was found to be metabolised to five times higher concentrations of its monoester MEP, which could also be a result of the above mentioned migration of DEP-containing adjacent wells.

Our study showed that cytokine secretion by innate immune cells as well as by T cells was influenced by phthalates. Some of the cytokines secreted by PHA-P-stimulated MNC, namely IL-6, IL-10 and TNF-α, may also originate from monocytes/macrophages. Thus, it cannot be excluded that the observed influence may be due to a phthalate-mediated effect on monocytes/macrophages only.

The cytokine secretion patterns in stimulated MNC exposed to either DEP or DnBP were similar, suggesting but not proving a common mechanism of action. On the other hand, MnBP seemed to act differently, especially in PHA-P-stimulated MNC, which may indicate that a separate signal pathway is involved. In LPS-stimulated MNC, only TNF-α secretion differed in MNC exposed to MnBP, compared to those exposed to DnBP or DEP. While MnBP enhanced TNF-α secretion, the latter two compounds inhibited secretion of this cytokine. IL-6, CXCL8 and IL-10 were influenced to a slightly lesser degree in MnBP-exposed cell cultures, compared to cultures stimulated with DnBP or DEP (S1 Table, estimated differences/ratios in Tukey’s Post hoc analysis). This suggested that MnBP was less potent than the diesters. Kristensen et al. have previously measured intracellular phthalate metabolite contents in a mouse sertoli cell line and found that DnBP but not MnBP entered the cells and influenced PG synthesis [22]. In the present study, phthalate metabolite content was only assessed in cell supernatants. Thus, it is unknown if the phthalate-uptake by MNC also differentiates between di- and monoesters. In summary, compared to its diester DnBP, the monoester MnBP might act through a separate signal pathway and/or is less potent due to a limited uptake by MNC.

As their structure resembles that of PGs [25], phthalates have been suspected to influence PG signalling and/or synthesis. PGs on the other hand, have been reported to induce a cytokine secretion pattern similar to that observed in phthalate-exposed monocytes/macrophages [21,37,38]. All three studies demonstrated an inhibition of TNF-α production by Prostaglandin E_1_ (PGE_1_), PGE_2_ or PGE_3_, with a simultaneous increase of either IL-6 [21] or IL-10 production [37,38]. Thus, if the observed influence of phthalates on cytokine production was due to an increase in PG production, or was caused by enhanced PG-receptor signalling, an increase in cAMP production would be expected, as PG is assumed to exert its effects via second messenger cAMP [21]. However, no cAMP was detectable in supernatants from LPS-stimulated MNC, neither in controls nor phthalate exposed cells. Two not mutually exclusive explanations, may account for this: The influence of phthalates was not mediated through PG or cAMP, or the cAMP assay used in this study was not sensitive enough to measure the secreted cAMP. The latter is likely, since the cAMP levels found by Bailly et al. were below or very close to the lowest standard used in our study [21]. However, cAMP in the present study was assessed in the supernatant, thereby investigating an accumulated response, rather than an immediate response when measured intracellular, as was done by Bailly et al. [21]. The method used in our study has been evaluated in human thyroid cell cultures [39], and changes in intracellular cAMP levels corresponded well to changes in extracellular cAMP levels [40]. The accumulated response (i.e. extracellular cAMP levels) are however expected to be higher than that of the immediate intracellular response, and we should have been able to detect cAMP in the present study.

Another explanation for the observed influence of phthalates on cytokine secretion relates to their potential toxicity. Thus, phthalate-induced loss of membrane integrity may have resulted in leakage of preformed cytokines into the supernatants. Using LDH release as a marker of cell death, we found that only LPS-stimulated MNC exposed to 100 μM DEP had clearly higher LDH levels than those of the ethanol control. However, the cytokine secretion pattern from DEP-exposed MNC was similar to that of DnBP-exposed MNC, suggesting that no cytokines were leaked after exposure to DEP. Moreover, results from PHA-P-stimulated MNC suggested that none of the phthalate-exposed MNC released more LDH than the ethanol control.

Phthalates in this study were dissolved in ethanol and thus, experiment wells and vehicle controls contained 1‰ (v/v) ethanol. Ethanol itself increased IL10 and IL-1β secretion from LPS stimulated MNC compared to the medium control, indicating that ethanol itself has the ability to influence cytokine secretion. Thus, the observed influence by phthalates might have been caused by an interaction with ethanol. To further elucidate this, the effect on cytokine secretion by DMSO dissolved phthalates could be investigated, to see if the same change in cytokine secretion pattern is found.

In LPS stimulated MNC, the investigated phthalate diesters enhanced secretion of both inflammatory and anti-inflammatory cytokines (IL-6, CXCL8, IL-10), and inhibited secretion of a strong pro-inflammatory cytokine (TNF-α). In PHA-P stimulated MNC, the diesters inhibited cytokines with predominantly inflammatory abilities which also are import for directing cell differentiation of cells belonging to the adaptive immune system. Clinical consequences of this could be a changed or even weakened immune response to pathogens. A recent epidemiological study by Grandjean et al. on perfluorinated compounds (PFC) in serum of children from the Faroe Islands has found a negative association between PFC and antibody concentrations against diphtheria toxoids [41], an indication that the efficiency of childhood immunisation is reduced by PFC. In an *in vitro* study by Corsini et al. on cytokine secretion both LPS and PHA exposed human MNC demonstrated that PFC were able to influence the cytokine pattern of these cells. PFC inhibited TNF-α and IL-6 but not IL-10 secretion from LPS-stimulated MNC and IL-4, IL-10 and INF-γ secretion from PHA-stimulated MNC [42]. Except for IL-10 secretion and other cytokines that were not assessed by Corsini et al., the cytokine secretion pattern is similar to the one found in the present study with phthalates. If this change in cytokine secretion pattern by phthalates could result in a diminished immune response in connection with childhood immunisation will need further investigation *in vivo*.

## Conclusion

DEP and DnBP are metabolised by human MNC to their respective monoester. They influence cytokine secretion from both monocytes/macrophages and T cells. Even though DEP resulted in a release of LDH by monocytes, the altered cytokine secretion pattern did not seem to be a result of cell death. It is therefore likely that the phthalate diesters are taken up and metabolised by MNC, and that they influence cellular signal pathways that govern cytokine production. Measurements of intracellular phthalate metabolites should be able to confirm this. The phthalate monoester MnBP was not further metabolised to secondary metabolites. Nonetheless MnBP influenced cytokine secretion but not to the same extent as that of the diesters.

## Supporting Information

S1 Table LPS-stimulated MNC exposed to phthalates: results from statistical analysis. For some cytokines, log-10 transformed data were used in the statistical analysis, thus the estimated differences are relative, i.e. ratios. For example: The mean IL-6 secretion influenced by DEP 0.1 μM was estimated 20% higher than the mean IL-6 secretion in the control group, and lies with 95% certainty between 21% below and 48% above the mean of the control group. Red coloured numbers: p<0.05. CI: confidence interval. ^†^estimated difference given as a ratio.
(XLSX)

S2 Table PHA-P-stimulated cells exposed to phthalates: results from statistical analysis. For some cytokines, log-10 transformed data were used in the statistical analysis, thus the estimated differences are relative, i.e. ratios. Red coloured numbers: p<0.05. CI: confidence interval. ^†^estimated difference given as a ratio.
(XLSX)
